# A contemporary analysis of the pre- and intraoperative recognition of multigland parathyroid disease

**DOI:** 10.1007/s00423-023-03087-w

**Published:** 2023-10-09

**Authors:** E Lawrence, G Johri, R Dave, R Li, A Gandhi

**Affiliations:** 1 Wythenshawe Hospital and Nightingale Breast Cancer Centre, Manchester University Foundation Trust, Southmoor Road, Manchester, M23 9LT UK; 2 Division of Cancer Sciences, Faculty of Biology, Medicine and Health, University of Manchester, Oglesby Cancer Research Building, M20 4GJ, Manchester, UK

**Keywords:** Sporadic primary hyperparathyroidism, Multigland parathyroid disease, Discordant parathyroid imaging, Subtotal parathyroidectomy

## Abstract

**Background:**

Despite advances in biochemical and radiological identification of parathyroid gland enlargement, primary hyperparathyroidism (PHPT) due to sporadic multigland parathyroid disease (MGPD) remains a perioperative diagnostic dilemma. Failure to recognise MGPD pre- or intraoperatively may negatively impact surgical cure rates and result in persistent PHPT and ongoing patient morbidity.

**Methods:**

We have conducted a comprehensive review of published literature in attempt to determine factors that could aid in reliably diagnosing sporadic MGPD pre- or intraoperatively. We discuss preoperative clinical features and examine pre- and intraoperative biochemical and imaging findings concentrating on those areas that give practicing surgeons and the wider multi-disciplinary endocrine team indications that a patient has MGDP. This could alter surgical strategy.

**Conclusion:**

Biochemistry can provide diagnosis of PHPT but cannot reliably discriminate parathyroid pathology. Histopathology can aid diagnosis between MGPD and adenoma, but histological appearance can overlap. Multiple negative imaging modalities indicate that MGPD may be more likely than a single parathyroid adenoma, but the gold standard for diagnosis is still intraoperative identification during BNE. MGPD remains a difficult disease to both diagnose and treat.

## Introduction

Recently, the World Health Organisation (WHO) has proposed a new nomenclature for hyperparathyroidism [1]. For sporadic primary hyperparathyroidism in which more than one gland is autonomously secreting excess PTH the term multiglandular parathyroid disease (MGPD) is recommended. This clinical entity is now considered to be a germline susceptibility-driven neoplastic diagnosis composed of several synchronous or metachronous proliferations, rather than true hyperplasia [1, 2]. Within this new nomenclature the entity previously known as parathyroid hyperplasia is reserved for multiglandular hyperparathyroidism secondary to chronic renal failure (so called secondary hyperparathyroidism) [1].

The only curative option for PHPT is parathyroidectomy. Traditionally bilateral cervical exploration was favoured for visualisation of all four glands and resection of hyperfunctioning parathyroid(s) [3]. With advances in preoperative localisation techniques and availability of intraoperative PTH measurements, explorations may be undertaken as a focussed, minimally invasive procedure, with comparative patient outcomes [3]. Successful focussed parathyroidectomy can reduce operative time, hospital length of stay and healthcare costs [4]. However, one potential problem with parathyroidectomy, either focussed or bilateral cervical exploration, is that failure to identify and treat MGPD, preoperatively and/or intraoperatively, can result in continuing hyperparathyroidism. The optimal approach to diagnosis and management of MGPD remains controversial and currently there are no specific guidelines towards its management. The preoperative identification of MGPD and intraoperative surgical management of primary hyperparathyroidism due to MGPD will be the focus of this review. Parathyroid hyperplasia secondary to renal disease and parathyroid dysfunction as a result of genetic disorders (such as Multiple Endocrine Neoplasia) are outside the scope of this article.

## Methods

We conducted a systematic literature search of studies published in English on sporadic multiglandular parathyroid disease and PHPT between 1 January 1983 and 30 September 2022. PubMed, Scopus and the Cochrane database were explored. Additionally, references detailed in practise guidelines, meta-analyses and review articles were manually searched. Case reports, case series with less than ten subjects and studies on PHPT which did not report on the clinical/surgical/biochemical profile were excluded. Eighteen randomised clinical trials, eight systematic reviews and meta-analyses and twenty-seven observational studies were included after all authors agreed upon all selected articles.

### Physiology of PTH secretion

PTH secretion is mediated by the calcium sensing receptor (CaSR) signalling pathway. CaSR is located on the cell surface of parathyroid chief cells. In the presence of circulating calcium, CaSR is activated and phosphorylated [5] suppressing PTH secretion via two mechanisms reduced PTH production and by overexpression of vitamin D receptors on parathyroid chief cells, thereby increasing their sensitivity to the negative feedback exerted by 1,25(OH)2-vitamin D, further suppressing PTH production [6]. There is a sigmoidal positive dose-response relationship between extracellular and intracellular calcium and a similar but inverse relationship with PTH secretion [7]. PTH stimulates osteoclast-mediated bone resorption, renal reabsorption of calcium and 1,25(OH)2D synthesis to increase calcium levels. Rising circulating calcium levels provoke a negative feedback loop reactivating CaSRs, signalling pathways and preventing PTH secretion.

### Pathophysiology of PTH secretion in hyperparathyroidism

In primary hyperparathyroidism, the nuanced relationship between extracellular calcium concentration and PTH secretion is lost. The sigmoid calcium-PTH curve is in effect shifted to the right as a result of parathyroid cells secreting more PTH at any given level of calcium [7, 8] and diseased hypersecretory cells developing a new “set point” to maintain serum Ca^2+^ concentration at a constant, but supranormal level [8].

### Histopathology of multiglandular parathyroid disease in PHPT

The normal parathyroid appears grossly as a tan coloured, well circumscribed ovoid tissue of variable measurement, up to 6–8 mm, and weighing between 40 and 60 mg each [6]. Parathyroid glands larger than these measurements may be considered pathological [1]. There is no exact consensus of size or weight which differentiates parathyroid adenomata from the hyperplastic proliferative changes seen in MGPD [9, 10].

The predominant cell type within normal parathyroid glands are chief cells producing PTH. The remaining cells are oxyphil cells, rich in mitochondria and transitional cells representing an intermediate phase between chief and oxyphil cells [11]. Histological examination of parathyroid glands in MGPD reveals a gross increase in parathyroid parenchymal cell mass with a nodular proliferation of parathyroid chief cells and other cell types [12]. The cut surface may reveal nuclear pleomorphism and atypia as well as degenerative features [12]. The glands will typically lack a rim of normal or atrophic parathyroid tissue commonly seen in adenomata.

Histologically, it can be difficult to distinguish cellular proliferation seen in MGPD from adenoma. Features supporting the former include involvement of more than one gland, no clear capsule, fewer fat cells and absence of a normal appearing peripheral parathyroid tissue rim around the excised gland [6, 11, 12]. However, there is considerable overlap in characteristics compared to adenoma and currently no clearly defining histological criteria exist.

## Preoperative identification of multiglandular parathyroid disease

### Symptomatic vs asymptomatic PHPT

Traditional symptoms of PHPT are well recognised and consist of nephrolithiasis, bone loss (particularly cortical bone), gastrointestinal and psychiatric disturbances. Most symptomatic patients with PHPT display a more nebulous array of nonspecific symptoms such as fatigue and cognitive decline [13]. These symptoms can be subtle and long standing, leading patient and clinician to consider the patient “asymptomatic”. However, some argue that PHPT is uncommonly truly asymptomatic [14].

Unfortunately, the presence or absence of symptoms does not allow differentiation between single gland adenoma and MGPD [15]. In those patients who do display symptoms there is no difference in presenting symptomatology between patients who have MGPD and parathyroid adenoma [10]. Thus, these criteria cannot be used for surgical planning.

### Preoperative parathyroid hormone and calcium levels in MGPD vs single gland disease

Preoperative levels of parathyroid hormone and serum calcium are positively linked to the size and weight of hyperfunctioning parathyroid tissue in many [9, 16–18] but not all [19, 20] studies. However, examining the relative sizes of parathyroid glands removed for single gland disease versus MGPD has not shown a consistent difference in size of excised gland(s) nor preoperative parathyroid hormone levels [21, 22]. Therefore, preoperative levels of parathyroid hormone and calcium cannot be reliably used to predict MGPD [9].

### Preoperative imaging of parathyroid glands in MGPD

Localising imaging techniques are an imperative preoperative assessment for surgeons planning minimally invasive parathyroidectomy to avoid bilateral neck exploration (BNE). Parathyroid glands can be visualised using a range of modalities, including ultrasound (USS), sestamibi scanning, computed tomography (CT), magnetic resonance imaging (MRI) and positron emission tomography scans (PET) [23]. USS is an established performed first-line imaging technique being widely available and cost-effective. Normal parathyroid glands are not typically visible on USS due to their size therefore, a visible parathyroid gland, identified as an enlarged hypoechoic ovoid structure posterior to the thyroid gland [24], is suspicious of a pathological entity.

Colour doppler imaging may delineate the parathyroid from thyroid or lymph nodes. However, sensitivity is highly variable (60–71%) depending on operator experience, body habitus and presence of thyroid nodules Technetium-99 sestamibi scan and SPECT are widely accepted imaging studies with reported sensitivities ranging from 54 to 100% [25, 26], though the occurrence of false negative studies remains a problem [27]. Whilst combined sestamibi and USS can localise a single adenoma with a 95–99% success rate, preoperative localisation studies are often ineffective in their scope to provide differentiation between different aetiologies of hyperparathyroidism.

False negative imaging is frequent in MGPD with ultrasound and Technetium-99 labelled sestamibi scintigraphy. In one study of preoperative prediction of MGPD in 235 patients, ultrasound was negative in 96% with parathyroid MGPD compared to 12% of those with an adenoma. Furthermore, ultrasound was not able to identify MGPD in 23 patients with confirmed MGPD. Dual imaging was negative in 91% of MGPD compared to 2% of adenomas [10]. Shah et al. reported a 49% false negative rate, misdiagnosing MGPD for single gland disease with USS; dual imaging in patients with MGPD with USS and SPECT was concordant at 29%.

These studies infer that routine preoperative localisation can provide limited benefit in preoperative identification of MGPD. If preoperative localisation studies are negative, the pathological entity is more likely to represent MGPD compared to patients with one or more positive imaging results, where adenoma is far more commonly diagnosed [28].

The Wisconsin index and CaPTHUS score attempt to predict MGPD utilising both biochemical testing and imaging [29, 30]. The Wisconsin index combines assessment of preoperative serum calcium and PTH levels with intraoperative parathyroid gland weight therefore cannot be used as a predictor preoperatively. The CaPTHUS score includes serum calcium, PTH levels, urinary calcium and ultrasound and scintigraphy imaging. It can predict MGPD in those with positive imaging but is unreliable in patients with mild disease and low adenoma weight which are more common presentations of MGPD [15, 31]. Whilst useful, [32], both the Wisconsin index and CaPTHUS score have been found to have variable sensitivity, specificity, positive and negative predictive values [33–36] and thus have not found universal acceptance and do not feature in national guidelines on the surgical management of hyperparathyroidism [37–39].

A retrospective study of 2185 patients found that 38.3% of patients had negative preoperative imaging and these patients had smaller parathyroids by size and mass, higher incidence of MGPD (12.8% vs 5.4%), and lower incidence of single adenoma (73.6 vs 86.0%) compared with localised patients. There was no significant difference in cure rates (96.2% vs 97.7%) [40]. More recently, a retrospective, single centre study of 549 patients aimed to determine if MGPD could be determined preoperatively. In their series, 38 (7%) of patients had MGPD and these patients were more likely to have negative sestamibi, ultrasound or both (92% vs 6%; 96% vs 4%; 91% vs 2%) [10]. These studies confirm that patients with negative preoperative imaging are more likely to have MGPD. However, positive imaging localisation preoperatively does not negate the possibility of multigland disease.

4-Dimensional Computed Tomography (4D-CT) amalgamates changing patterns of enhancement of parathyroid tissue over time (the so-called fourth dimension) with standard three-dimensional computed tomography. It has the benefits of a short imaging time and high spatial resolution but the downside of a 57-fold higher dose of ionising radiation compared to sestamibi CT scanning [41]. Its’ accuracy depends on the number of CT phases performed with debate as to the optimum number of phases that should be used to improve accuracy without significantly increasing radiation exposure [42]. Though 4D-CT is a proven useful technique for diagnosing parathyroid adenomas, these benefits are not extended to cases of MGPD where it is shown to have low sensitivity and specificity [43]. Initial promise shown by combining 4D-CT scanning with biochemical data in predicting MGPD [44] has not been confirmed by subsequent studies [45].

Parathyroid MRI is a relatively new modality with recent studies showing excellent sensitivity and specificity (up to 97.8% and 97.5%, respectively) in the detection of parathyroid adenomata [46, 47]. Importantly, MRI has also been shown to demonstrate reliable accuracy in multiglandular disease with 8/8 enlarged gland and 6/7 ectopic glands detected in a series reported by Agiro et al. [46], more studies are needed to validate these findings. The current consensus is that 4D-CT and MRI are reserved as second line imaging, if ultrasound and sestamibi have failed to localise the pathology.

Selective venous sampling is a highly invasive, but useful adjunct investigation generally reserved for patients with persistent or recurrent primary hyperparathyroidism following attempted parathyroidectomy. The only published meta-analysis on this investigation included 12 studies concluding that though venous sampling had higher sensitivity compared to non-invasive modalities in redo parathyroid surgery, its invasiveness precluded its routine use for preoperative localisation [48].

## Surgical considerations

### Use of preoperative imaging to guide operative approach

Intraoperative, visual assessments of size, colour and weight are inconsistent predictors of histological diagnosis [49] and therefore, it is difficult for the operating surgeon to distinguish MGPD clinically. This can result in the potential for incomplete resection of pathological parathyroids.

Traditional surgical management of PHPT consists of bilateral neck exploration (4 gland exploration), with successful identification and excision of diseased parathyroid glands conferring biochemical cure rates of over 95% in specialised centre [50, 51]. BNE is recommended where imaging has failed to identify a solitary adenoma, or is discordant [52]. Preoperative imaging must also be considered in context with the patient’s clinical history. For example, family history or childhood history of irradiation and/or lithium use should raise suspicion of multiglandular disease [53, 54].

A recent study of 1158 patients evaluated the success of limited exploration for primary hyperparathyroidism using ultrasound, sestamibi and intraoperative parathyroid hormone [55]. All patients initially underwent limited exploration based on imaging localisation, then regardless of results had bilateral exploration performed at the same operation to identify the presence of additional parathyroid pathology. A single abnormal gland was suspected on sestamibi in 74%, and ultrasound in 80% with concordant imaging in 64% of patients. However, unsuspected multiglandular disease was identified at bilateral exploration in 22%, 22% and 20% of these patients, respectively.

### What is the appropriate operative approach for multiglandular parathyroid disease?

Traditionally, MGPD has been managed by subtotal parathyroidectomy, leaving a viable fragment of parathyroid tissue roughly the size of normal parathyroid gland (approximately 50 mg) in situ [56]. Other described methods include parathyroidectomy with auto transplantation of parathyroid tissue or total parathyroidectomy with replacement treatment (calcium and calcitriol), although the latter approach is described more in familial aetiology of PHPT [56, 57]. Between these different methods of parathyroidectomy, a subtotal resection is more commonly utilised due to comparable rates of disease persistence, recurrence and a lower risk of postoperative hypoparathyroidism [54].

Paloyan et al. [58] published long term results in 292 consecutive patients who underwent subtotal parathyroidectomy for primary hyperparathyroidism and found that 97.6% were cured after their first operation. A more recent study compared whole vs partial gland remnant for 172 patients undergoing subtotal parathyroidectomy; the study was not randomised but rather an intraoperative decision was made by the surgeon based on the size of the parathyroid glands and clinical context. The overall cure rate was 97.1%, with temporary hypocalcaemia in 27.3% and permanent hypocalcaemia in 0.6%; there was no significant difference in disease persistence, recurrence or temporary/permanent hypocalcaemia between the two groups [56]. A large international audit of 5861 patients with sporadic PHPT [59] found that 26.9% underwent BNE, 4.6% underwent thymectomy and autotransplantation of parathyroid tissue was performed in 2.5%. Of all PHPT patients, those who underwent BNE had a higher rate of persistent hypercalcaemia (7.3%) compared with those who had unilateral exploration (3.3%) or focused operations (3.1%), but these findings are almost certainly reflect underlying pathology rather than surgical technique. For patients with PHPT caused by sporadic MGPD, BNE subtotal parathyroidectomy should be the operation of choice. However, there is no clear consensus as to how much parathyroid tissue should be left in situ.

### Intraoperative recognition

Confirming the presence of parathyroid tissue during specimen resection may be aided by several factors. According to the consensus report of European Society of Endocrine surgeons, the observed size of the parathyroid is an indicator of hypersecretion and all visually enlarged parathyroid glands are typically excised. Weighing and measuring the specimen after removing surrounding fat, lymph or other tissue can delineate between adenoma and carcinoma, or from normal tissue [60, 61]. Smaller resected glands (<200 mg) have also been associated with higher risk of persistent disease [31, 62]. Shah et al. presented a single centre series of 1890 consecutive cases of PHPT, of which 13.4% were found to be MGPD. Of these, 96.1% were diagnosed intraoperatively and 3.9% postoperatively. Intraoperative diagnosis was prompted by intraoperative PTH monitoring (IOPTH) in 38.5%, surgeon interpretation of imaging in 38.1%, observing ipsilateral gland enlargement in 11.0%, by finding an initial gland <200 mg in 10.3% and 2.0% had unexpected MGPD during thyroidectomy [63]. In addition, there is a positive correlation between preoperative PTH levels and parathyroid gland weight for single gland disease [16]. However, there is a high degree of variability in parathyroid gland size and it alone is not a reliable indicator of adenoma or MGPD [16, 49].

### Intraoperative PTH monitoring

Intraoperative parathyroid assay may help ensure complete removal of hyperfunctioning parathyroids by distinguishing adenoma from MGPD [64]. Sensitivity has been reported as nearly 95%, where a reduction in PTH levels of over 50% following resection of a solitary gland can confirm adenoma [65]. The Rome criteria has been demonstrated the most useful in intraoperative detection of MGPD, and the Miami criteria the highest accuracy in intraoperative prediction of cure [66]. Rapid intraoperative PTH assay can be a useful adjunct to surgery if interpreted correctly but may be associated with a high rate of false positives [67].

### Postoperative outcomes in patients with MGPD

For patients with sporadic PHPT due to MGPD, the overall cure rate is generally reported as above 95% in those undergoing a subtotal parathyroidectomy [38, 56]. Transient hypocalcaemia occurs in 15–30% following parathyroidectomy and larger parathyroid glands are also associated with higher rates of postoperative hypocalcaemia [68]. Overall, permanent hypoparathyroidism in patients undergoing surgery for PHPT is rare with a reported incidence of 0–0.5%, however, it is significantly higher in patients with MGPD undergoing subtotal parathyroidectomy with at 10–15% [54, 69].

## Conclusion

Sporadic MGPD involving all four parathyroid glands is a common cause of PHPT. Although less common than parathyroid adenoma, it remains a challenge for clinicians due to the inability to definitively diagnose preoperatively. A thorough review of the literature was unable to identify any preoperative clinical factors or investigations that could reliably determine a diagnosis of MGPD. Biochemistry can provide diagnosis of PHPT but cannot reliably discriminate parathyroid pathology, particularly between adenoma and MGPD. Histopathology can aid diagnosis between MGPD, adenoma and carcinoma, but histological appearance can overlap, thus resulting in difficulty with accurate diagnosis. Multiple negative imaging modalities indicates that MGPD may be more likely than a single parathyroid adenoma but the gold standard for diagnosis is still intraoperative identification during BNE. IOPTH assays are an option to distinguish pathological parathyroid tissue, as well as gross visual examination and weight. MGPD remains a difficult disease to both diagnose and treat and the literature, to date, does not yield any reliable techniques to achieve either aside from traditional BNE and intraoperative identification.
